# Disability among Older People: Analysis of Data from Disability Surveys in Six Low- and Middle-Income Countries

**DOI:** 10.3390/ijerph18136962

**Published:** 2021-06-29

**Authors:** Josephine E. Prynn, Sarah Polack, Islay Mactaggart, Lena Morgon Banks, Shaffa Hameed, Carlos Dionicio, Shailes Neupane, GVS Murthy, Joseph Oye, Jonathan Naber, Hannah Kuper

**Affiliations:** 1 International Centre for Evidence in Disability, Clinical Research Department, London School of Hygiene & Tropical Medicine, London WC1E 7HT, UK; josephineprynn@gmail.com (J.E.P.); islay.mactaggart@lshtm.ac.uk (I.M.); morgon.banks@lshtm.ac.uk (L.M.B.); Shaffa.Hameed@lshtm.ac.uk (S.H.); Hannah.kuper@lshtm.ac.uk (H.K.); 2 UCL Institute of Cardiovascular Science, Gower Street, London WC1E 6BT, UK; 3 National Council on Disability, Guatemala City 01001, Guatemala; dr.carlosdionicio@gmail.com (C.D.); jonathan@rompglobal.org (J.N.); 4 Valley Research Group, Kathmandu 44600, Nepal; shailes@varg.wlink.com.np; 5 Indian Institute of Public Health, Hyderabad 122002, Telangana, India; GVS.Murthy@lshtm.ac.uk; 6 Sightsavers Cameroon, Yaoundé, Cameroon; joye@sightsavers.org; 7 Range of Motion Project, P.O. Box 100915, Dever, CO 80250, USA

**Keywords:** disability, older adults, participation, COVID-19

## Abstract

This analysis of surveys from six low- and middle-income countries (LMICs) aimed to (i) estimate the prevalence of disability among older adults and (ii) compare experiences and participation in key life areas among older people with and without disabilities which may show vulnerability during the COVID-19 pandemic. Data were analysed from district-level or national surveys in Cameroon, Guatemala, Haiti, India, Nepal and the Maldives, which across the six databases totalled 3499 participants aged 60 years and above including 691 people with disabilities. Disability was common among adults 60+, ranging from 9.7% (8.0–11.8) in Nepal to 39.2% in India (95% CI 34.1–44.5%). Mobility was the most commonly reported functional difficulty. In each setting, older people with disabilities were significantly less likely to be working and reported greater participation restrictions and environmental barriers in key life areas compared to people in the same age categories without disabilities (*p* < 0.05). Disability is common in this population, and older people with disabilities may have greater difficulties participating in COVID-19 responses and have high economic vulnerabilities. It is imperative to prioritise the needs of older people with disabilities in the COVID-19 pandemic, including ensuring accessibility of both health services and the community in general.

## 1. Introduction

The world’s population is ageing, particularly in many LMICs [1]. By 2050, it is predicted that 80% of people aged 60 and over will live in LMICs [1]. While high-income countries have had many decades to adjust to an ageing population, this process is happening much more rapidly in many LMICs, with the proportion of the population aged over 60 set to double from 10% to 20% in just over 20 years in countries such as Brazil, China and India [1]. The number of older adults is increasing most rapidly in sub-Saharan Africa (SSA), where the population of older people is expected to more than triple between 2015 and 2050 [2].

In the context of the ongoing COVID-19 pandemic, older people, and those with underlying health conditions, have the highest morbidity and mortality risk [3]. They are also at risk of being left behind in the responses to COVID-19 and in economic rebuilding when the pandemic is resolved [4]. Older people are not a homogeneous group, and vulnerability during the COVID-19 pandemic, as well as in general, will vary by different characteristics. One group who are likely to be particularly marginalised, however, are older people with disabilities. Despite this, evidence relating to older people with disabilities is lacking, particularly in LMICs.

Disability and age are closely linked: as people get older, the prevalence of disability increases [1,5]. Disability is conceptualised by the World Health Organisation (WHO) International Classification of Functioning, Disability and Health (ICF). A health condition (e.g., arthritis) can impair body functions and structures (e.g., joints), limit activities (e.g., reduced mobility) and restrict participation (e.g., reduced social engagement). This effect is mediated by both environmental and personal factors (e.g., social support, availability of assistive devices)**.** Therefore, to understand how disability is affecting older people in different contexts, we must examine barriers to activity and participation as well as the presence of impairment. Participation restrictions among older people can have a significant negative impact on the well-being of the individual, as well as limiting the significant contributions older people make to the welfare of their families and wider society, including remaining economically active until late in life and caring for grandchildren [6]. While disability in older age has been relatively well-characterised in high-income countries (HICs) [7,8,9,10], there are very few data from LMICs, where the landscape is likely to be very different due to the disparity in availability and quality of healthcare interventions, particularly for chronic conditions [11,12,13].

Both older people [1] and people with disabilities [5] face discrimination and exclusion, and this may be experienced as a double discrimination by older people with disabilities. However, there has been limited research into this area. It is critical to understand the experiences of older people with disabilities to ensure that health and social policies are developed with their needs in mind, particularly during the current COVID-19 pandemic [14]. Therefore, this paper, through secondary analysis of population-based disability surveys from six different LMICs, aims to assess (i) the prevalence of disability in older people in a range of LMICs and (ii) describe sociodemographic characteristics and experiences of participation in key life areas by older people with disabilities.

## 2. Materials and Methods

We conducted secondary data analysis of six cross-sectional population-based surveys of disability from Cameroon, Guatemala, Haiti, India, the Maldives and Nepal. These surveys also assessed the living situation of people with and without disabilities, including work, poverty, health and participation. The surveys included people of all ages, but our analysis focused exclusively on adults aged 60 and older (the age cut-off used by the WHO to define older people) [3]. All the surveys were conducted by the International Centre for Evidence in Disability (ICED) at the London School of Hygiene & Tropical Medicine (LSHTM) in collaboration with national and international partners and approved by relevant ethics committees. Details of the datasets included in the study are summarised in Table S1, and full methods have been published previously [15,16,17,18,19]. Members of Disabled Persons Organisations were involved in the design, dissemination and reporting of the research in some settings.

### 2.1. Sampling

All studies used two-stage cluster sampling. In the first stage, a predetermined number of clusters (e.g., villages, enumeration areas) were selected using probability proportional-to-size sampling (PPS), using the most recent country census as a sampling frame. Next, modified compact segment sampling was used to select households within clusters: using a map of the cluster (either drawn in collaboration with local leaders or using pre-existing maps where available) each cluster was divided into equal segments of the defined segment sample size (e.g., approximately 80 people in Cameroon, 50 people in Guatemala). One segment was selected at random for inclusion; all households within the selected segment were visited door-to-door, and all consenting household members were recruited until the sample size was reached. Participants who were not available on first visit were visited at least two further times to maximise the response rate. For example, in Cameroon, the 2005 Census was used as a sampling frame from which clusters (census enumeration areas) were selected using PPS. Using maps, each cluster was divided into segments of around 80 people. One segment was then selected at random for inclusion: enumerators visited each house within the segment door-to-door until 80 eligible participants had been recruited. Sampling procedures for each setting are provided in Table S1.

### 2.2. Prevalence of Disability

In Cameroon, Guatemala, India, the Maldives and Nepal, disability was assessed using the Modified Washington Group Extended Set of questions on functioning [20]. This tool asks about the level of difficulty (“no difficulty”, “some difficulty”, “a lot of difficulty” or “can’t do at all”) a participant has in the following domains: seeing, hearing, walking or climbing steps, communicating (understanding/being understood), remembering or concentrating, self-care, upper body strength and fine motor dexterity. It also includes questions on frequency and severity of experiencing symptoms of anxiety and depression. For this analysis, disability was defined as experiencing “a lot of difficulty” or more in any of the above domains or reporting “a lot” of anxiety/depression daily. Participants were directly interviewed where possible. For people unable to communicate independently, a caregiver/family member was interviewed as a proxy. In Nepal, proxy respondents were also interviewed if the participant was unavailable at time of survey team visit, and in the Maldives, a proxy respondent was interviewed if the participant was unavailable after at least three return visits by an interviewer. We did not estimate prevalence from Haiti as this used the Washington Group Short Set of Questions (which asks about difficulty in only six domains: seeing, hearing, walking, self-care, communication and cognition) through proxy report by head of household, which limits comparability.

### 2.3. Comparison of Older People with and without Disabilities

A case-control study was nested within each of the surveys to compare people with and without disability in key life areas. ‘Cases’ were defined in the same way as in the prevalence analysis, described above. In Haiti, cases were defined as participants reporting “a lot of difficulty” or “can’t do at all” on one domain or “some difficulty” in more than one domain (see Table S1).

In each setting, one control (person without disabilities, according to above criteria) was recruited for each case, matched by age, sex and cluster where possible. The specific matching criteria varied by setting (Table 1), and exact matching of one control for each case was not always possible. In Guatemala, Haiti and the Maldives, there were more controls than cases. This is because, due to the high prevalence of disability in this older age group, it was not always possible to identify an age- and sex-matched control without a disability from the same cluster. In India and Cameroon, there were more controls than cases included in the analysis. This is because the data are from a wider study which also included ‘cases’ with clinically assessed vision, hearing and mobility impairment and matched controls. For the current analysis, we included all the ‘controls’, but, for comparability with the other surveys, only ‘cases’ who met the above study definition using the Washington Group questions were included. Participants were interviewed about sociodemographic indicators, work, education, health, water and sanitation, participation restrictions and environment.

### 2.4. Other Variables

Principal component analysis (PCA) was used to categorise household-level socioeconomic position (SEP) into four groups within each site, based on the ownership of assets and building materials of the home. Participants were classified as literate if they said they could read and write at least a little. Work was defined differently in different settings: in Cameroon and India it included work on a participant’s own land, but this was not included in the definition in other settings. Catastrophic health expenditure (collected in Nepal and the Maldives) was defined using the WHO definition of 25% or more of household income spent on healthcare [21]. In the settings with data on hypertension and diabetes diagnoses, this information was ascertained by self-report.

A participation score was calculated based on answers to questions asking about level of difficulty performing various activities in their current environment within the domains of self-care; domestic life; interpersonal life; major life areas (including school and occupation); and community, social and civic life. To characterize environmental barriers to participation, participants were asked how often a series of potential barriers were a problem for them and whether this was a “little problem” or a “big problem”. Environmental barriers were defined as a participant experiencing a “big problem” in any of the domains at least monthly. In Guatemala and the Maldives, participants were also asked about their quality of life using the WHO quality of life BREF instrument, which asks participants to rate, on a five-point response scale, their physical health, psychological factors, social relationships and environmental factors, as well as their overall quality of life and overall satisfaction with their health.

### 2.5. Statistical Analysis

Data were analysed using Stata (StataCorp, College Station, TX, USA). We calculated prevalence with 95% confidence intervals, using the ‘svy’ command in Stata to account for the sampling design. Logistic regression was used to compare sociodemographic factors, health indicators, health service, WASH access, work and difficulties with environment for older adults with and without disability (cases and controls) in each setting. Linear regression was used to compare participation scores. Conditional regression analyses were not undertaken, since there was not exact matching of cases and controls, so all regression analyses were adjusted for the matching variables (age and sex).

## 3. Results

### 3.1. Prevalence of Disability

As shown in Table 1 the prevalence of self-reported disability in adults aged 60 and over ranged from 9.7% (8.0–11.8) in Nepal to 39.2% in India (95% CI 34.1–44.5%). The prevalence increased with age to between 27.0% in Nepal (95% CI 19.1–36.6%) and 65.5% in India (95% CI 46.6–80.4%) in the 80+ age range. In each country, disability prevalence among the oldest adults (aged 80+ years) was at least double that of adults aged 60–69 years. The most commonly reported functional limitation domain in every setting was difficulty with mobility (Table 1).

### 3.2. Comparison of Older Adults with and without Disabilities

Overall, cases were more likely to be over-represented in the older age group (80+ years). This was because of the high prevalence of disability in this age group resulting in limited availability of eligible controls.

The comparison of sociodemographic between older people with disabilities (cases) and age–sex–location-matched controls without disability is shown in Table 2**.** There was no evidence of a significant difference in household socioeconomic position, literacy, education or marital status between older people with and without disabilities in any of the settings. The only exception was the Maldives, where older people with disabilities were more likely to belong to households in the wealthiest household socioeconomic group compared to people without disabilities (OR 2.6 95% CI 1.2–5.9) and people with disabilities were more likely to be illiterate (OR 2.4 95% CI 1.0–6.2).

Older adults with disabilities in were more likely to report having a serious health problem within the past 12 months compared to adults without disability, although this was only statistically significant in Guatemala (Table 3). The majority (>77%) of adults, both with and without disabilities, reported seeking healthcare if they had experienced a serious health issue. In Haiti, there was no difference in reported difficulty accessing care by disability status (OR 1.4; 95% CI 0.4–5.3). However, in Guatemala, older people with disabilities were 4 times more likely to report finding it difficult to understand the information given to them at health facilities (OR 4.1; 95% CI 1.3–12.7). In Guatemala and the Maldives (but not Nepal), older people with disabilities were significantly more likely to have been diagnosed with hypertension than those without disabilities, but this was not apparent for diabetes.

In the Maldives and Nepal, data were also collected on healthcare expenditures and household income with contrasting results (Table 3). In Nepal, households in which an older person with a disability was living were twice as likely to have spent more than a quarter of their income on healthcare (OR 2.1; 95% CI 1.0–4.7). In contrast, in the Maldives, households with an older person with a disability were less likely to have spent more than 25% of their income on healthcare (aOR 0.4; 95% CI 0.1–1.0).

In each country, older adults with disabilities were significantly less likely to have engaged in any work either in the past week or past year; for example, in Guatemala, 44% of older adults without disabilities had worked within the past week, compared to 18% of older adults with disabilities (OR 0.4, 95% CI 0.2–0.7).

**Figure 1:**
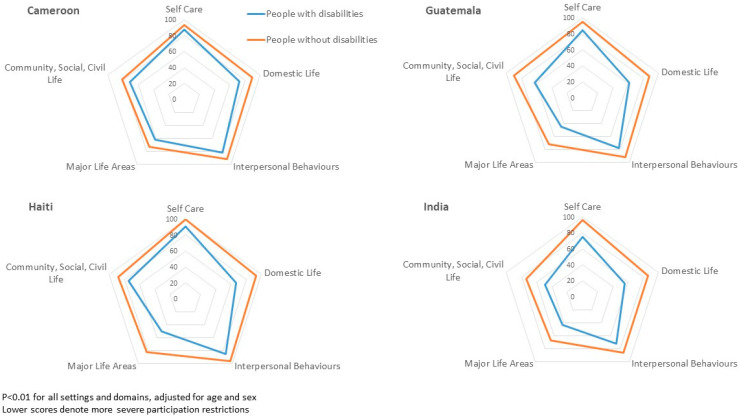
Comparison of participation scores among older adults with and without disabilities.

Older people with and without disabilities were compared in terms of key risk factors relevant to vulnerability to and from COVID-19 in Table 3. Questions about WASH access, considered with respect to sanitation facilities, were asked in Guatemala and Nepal; older people with disabilities were less likely to use the same sanitation facility as other household members, less likely to be able to use a sanitation facility without faecal contact and less likely to be able to use the facility without assistance from others; while these associations are not all statistically significant, the direction of association was consistent between the sites. In Cameroon, Guatemala, Haiti and India, participants were asked about their ability to perform a range of activities in their current environment (including with the assistance of any person or assistive device they used) in the areas of independent or supported self-care, domestic life, interpersonal behaviours, major life areas (work and education) and community or civic life. Figure 1 shows that mean participation scores in Guatemala, India, Cameroon and Haiti were consistently and significantly lower—indicating greater participation restrictions—for older people with disabilities than for those without. People with disabilities were also more likely to report that specific aspects of the environment, such as availability and accessibility of transport, the natural environment or the format of information, were a barrier to participation in the activities that mattered to them than people without disabilities, as shown in Table 4, but this was not always statistically significant. In India, lack of availability of assistance at home was a major barrier to participation. Quality of life was measured in Guatemala and the Maldives, and these scores were significantly worse among people with disabilities compared to those without in each of the subscales (overall health, physical health, psychosocial factors, social relationships and environmental factors; *p* < 0.01, data not presented). For the single question rating overall quality of life, scores were significantly worse for older people with disabilities compared to people without disabilities in the Maldives (*p* = 0.03), but not in Guatemala.

## 4. Discussion

The findings from six studies in different LMICs highlight that disability is common among older adults and that older people with disabilities are more likely than their peers without disabilities to face participation restrictions in key life areas and to report experiencing major health problems in the previous year.

Our findings align with existing evidence, although data on older people with disabilities are lacking from LMICs. The high prevalence of disability in older people is well known. The World Report on Disability suggests that approximately one in three people older than 60 have disabilities [5], and this is broadly consistent with our findings, with the exception of Nepal. Previous reports have also shown that people with disabilities have greater difficulties accessing WASH [22], have a greater vulnerability to poor health and have more difficulties accessing healthcare [23], although these analyses have not focussed specifically on older people. Poverty and disability are known to be closely linked, including among older people [24,25]. However, in our study, we saw no association between disability and household-level socioeconomic position (SEP) in any of the sites, with the exception of the Maldives where, surprisingly, people with disabilities were more likely to belong to households with higher socioeconomic position. A potential explanation for this lack of association is that cases with disabilities and controls were matched by location, and this may have weakened the relationship of poverty and disability, or that SEP was too blunt a tool to measure poverty in these settings. Further, asset-based measures are more reflective of long-term economic well-being and so may not reflect changes in wealth due to recent onset of disability [26]. However, our findings did show that older people with disabilities were less likely to be working than those without disabilities, which is consistent with the literature for the general population [5]. Participation restriction was experienced more by older people with disabilities than those without, in all studies that made these comparisons, in every activity domain. This finding is in keeping with multiple studies in high-income settings, which demonstrate that cognitive impairment, depression and difficulty with mobility are strongly associated with participation restriction among older adults [27,28,29].

The surveys analysed for this paper were all conducted prior to the COVID-19 pandemic. However, there are a number of findings that deserve consideration in terms of their implications in the context of the ongoing pandemic. Our findings support increasing evidence that older people with disabilities are particularly vulnerable during the COVID-19 pandemic, both in terms of risk of morbidity and mortality and also to suffering adverse effects of control measures [14,30]. First, disability increases with age, as does morbidity and mortality risk from COVID-19. Second, older people with disabilities may experience difficulties accessing WASH; were more likely to have reported a major health problem in the last year; and, in the Maldives and Guatemala, were more likely to have hypertension, which are all risk factors for COVID-19-related morbidity and mortality. Furthermore, older people with disabilities were less likely to have worked within the past week, which implies economic vulnerability during and in the aftermath of the pandemic. Older people with disabilities also reported more participation restriction in all major life areas due to multiple different environmental barriers; this may both limit engagement in COVID-19 response activities and increase the risk of isolation related to pandemic control measures. Together, these findings highlighth that older people with disabilities are likely to be particularly vulnerable to mortality from COVID-19 and to suffer economically during and after the pandemic [14,30]. Consequently, there needs to be a particular focus on older people with disabilities in the ongoing COVID-19 pandemic response.

Our findings also emphasise the importance of a COVID-19 response that is inclusive of older people with disabilities. The prevalence of visual, hearing and cognitive difficulties was relatively high in the surveys in this study, underscoring the need to provide information on COVID-19 (e.g., on reducing risk of coronavirus infection, vaccination campaigns) in accessible formats. Mobility was the most commonly reported functional limitation, highlighting the importance of physically accessible health services and vaccination sites so that older people with disabilities are not excluded. We also noted that older people with disabilities may face particular difficulties undertaking hygiene behaviours, and so appropriate, accessible hand-washing advice and facilities should be provided. Some older people with disabilities may be reliant on carers, which increases the challenges of preventing COVID-19, as social distancing may be more difficult, and the carer must have appropriate information. Furthermore, when considering older people with disabilities in LMICs, there is frequently an assumption that the traditional extended family unit will provide care. However, our study showed that in India, lack of availability of assistance at home was a major barrier to participation. Provision of care should therefore not be taken for granted, due to factors such as rapidly changing economies and societal norms over the past few decades that have resulted in increased access to formal employment, particularly for women who have traditionally been family caregivers, and the dispersal of many families [31].

Our findings also highlighted the barriers to the participation of older people with disabilities. There is a concern that this implies that they will be less engaged in preventive activities, but perhaps also less likely to receive (effective) care and treatment if they have COVID-19. Policies need to ensure that older people with disabilities are not deprioritised in treatment or discriminated against through protocols that create further barriers to their participation and that healthcare workers have training on disability awareness to understand the needs and rights of older people with disabilities. Furthermore, our results from Guatemala showed that older people with disabilities were more likely to find it difficult to understand the information given to them at health facilities, and this highlights that healthcare workers must have the skills to communicate with people with different impairment types.

Older people with disabilities also need to be supported to have their ongoing needs met during the pandemic. This study showed that older people with disabilities were more likely to have experienced a major health problem within the past year in each setting and so will require medication, specialist care, mental health support and other types of services. Furthermore, the social and economic impacts of COVID-19 may be particularly serious for older people with disabilities, as our findings showed that they were less likely to work and faced greater participation restrictions in self-care, domestic life, interpersonal behaviours and community life. It may therefore be important to identify older people with disabilities in communities to target them for additional support (e.g., food or emergency financial assistance). Continued health and well-being support in the pandemic may also require implementing new approaches, such as outreach services, tele-rehabilitation or providing additional carer support, all of which must adhere to infection control protocols. This should include mental health support services, considering that the prevalence of mental health conditions has increased as a result of the pandemic, social isolation and loneliness [32]. Finally, in the pandemic aftermath, there must be a focus on economic recovery for older people with disabilities, potentially through the provision of social protection or targeted livelihood interventions.

The need for inclusion of older people with disabilities is underlined by our findings but is also endorsed by the UN Convention on the Rights of Persons with Disabilities (Article 11) and the policies of many international actors. A number of principles can guide how this inclusion occurs. A twin-track approach is advocated, whereby people with disabilities are included in mainstream interventions (e.g., public health messaging) but also targeted with particular interventions (e.g., provided with accessible information). It is best to incorporate disability inclusion from the planning stage and include a dedicated budget. There must be consultation with older people with disabilities on their additional needs and potential solutions, and their participation should be encouraged in programming. Programming must be evidence-driven, and although this paper provides data on particular issues that need to be addressed, evidence is also needed on the effectiveness of different types of programming.

This study sheds light on the experiences of a particularly marginalised group: older people with disabilities in LMICs. Major strengths are that we have compared participation in key life areas of people with and without disabilities over a range of LMIC contexts. Furthermore, all the surveys were population-based and so are representative of their wider communities. A limitation of this study is that we used existing data, and so we were not able to assess all potential risk factors related to COVID-19; for instance, we focussed on access to sanitation as a proxy for access to hygiene, and data on household crowding, handwashing or access to information were not available. Another concern is that the definitions of cases for the case-control study varied somewhat between the different settings, which might have affected the strength of association between certain variables and disability. For example, in Haiti, this was based on the WG Short Set of Questions (a lot of difficulty in one domain, or some difficulty in at least two domains) reported by the head of the household. Further, the use of proxy responders, when a participant was unavailable, may have resulted in some underestimation of the prevalence in Nepal.

These studies were not specifically powered for comparisons between *older* people with and without disability, and therefore some caution is warranted in the interpretation of the findings, particularly in Haiti where the sample size was relatively small. Further, considering the disability definition used (self-reporting a lot of problems or cannot do with at least one functional domain), it is possible that some of the controls experienced mild forms of disability which may have diluted the association of disability and participation. There was a lack of existing data on older people with disabilities to allow comparison with our findings. Consequently, more evidence is needed, and data should be routinely collected disaggregated by age, sex and disability (including the disaggregation of disability data by age) in order to better understand the needs of this important and growing population. Recognition of the rights and needs of older people with disabilities is paramount and should be inherent within any governmental or organisational practice.

## 5. Conclusions

Older people with disabilities are an important and growing demographic in LMICs and so will increasingly have to be taken into account. They are likely to be particularly vulnerable during and in the aftermath of the COVID-19 pandemic. When planning health and social policy, including in the context of the current COVID-19 pandemic, it is imperative to prioritise the needs of older people, including ensuring accessibility of both health services and the community in general, as well as employing strategies to prepare for the certain increase in demand for support for older people in years to come.

## Figures and Tables

**Table 1:** Prevalence of disability overall and by age, sex and functional domain among older populations in Cameroon, Guatemala, India, Nepal and the Maldives.

	Cameroon	Guatemala	India	Maldives	Nepal
	(*n* = 429)	(*n* = 1148)	(*n* = 352)	(*n* = 449)	(*n* = 915)
	% (95% CI)	% (95% CI)	% (95% CI)	% (95% CI)	% (95% CI)
Overall prevalence of disability	24.9% (19.5–31.3)	22.4% (19.5–25.4)	39.2% (34.1–44.5)	29.4% (25.4–33.8)	9.7% (8.0–11.8)
Age					
60–69 years	15.0% (10.1–21.7)	15.6% (12.5–19.1)	29.9% (23.7–37.0)	19.0% (14.5–24.5)	8.1 (6.0–10.8)
70–79 years	28.9 (21.1–38.2)	22.7% (18.1–28.1)	55.6% (45.6–65.3)	40.5% (32.4–49.1)	6.8 (4.5–10.2)
80+ years	38.9% (28.4–50.5)	47.8% (39.3–56.5)	65.5% (46.6–80.4)	43.4% (32.8–54.7)	27.0% (19.1–36.6)
Sex					
Male	25.5% (18.6–34.1)	20.7% (17.3–24.5)	31.6% (25.6–38.3)	30.2% (24.5–36.7)	10.2% (7.7–13.5)
Female	24.5% (18.5–31.7)	23.9% (20.4–27.9)	46.4% (39.1–53.8)	28.6% (23.1–34.9)	9.3% (7.0–12.2)
Functional domain					
Seeing	9.3 (6.5–13.2)	9.2 (7.5–11.3)	13.6 (10.2–18.1)	7.8 (5.6–10.7)	1.9 (1.2–3.0)
Hearing	6.0 (0.4–10.5)	6.3 (4.8–8.2)	13.6 (9.7–18.8)	4.9 (3.2–7.3)	3.3 (2.3–4.7)
Mobility	14.0 (10.3–18.7)	13.7 (11.4–16.4)	19.3 (15.4–23.9)	21.4 (17.8–25.4)	4.9 (3.7–6.5)
Communication	0.5 (0.1–0.2)	2.3 (1.5–3.5)	2.6 (1.4–4.7)	3.8 (2.4–6.0)	2.3 (1.5–3.5)
Cognition	7.5 (5.9–11.1)	5.7 (4.3–7.6)	2.6 (1.2–5.3)	5.1 (3.4–7.6)	1.3 (0.7–2.3)
Self-Care	3.0 (1.7–5.3)	5.2 (4.1–6.6)	8.0 (5.3–11.6)	9.4 (7.0–12.4)	2.8 (1.9–4.1)
Upper body	4.4 (2.6–7.4)	7.8 (6.2–9.6)	10.5 (7.7–14.3)	9.8 (7.4–12.9)	2.5 (1.7–3.8)
Anxiety	3.2 (2.0–5.3)	5.5 (4.1–7.3)	7.4 (4.6–11.7)	3.1 (1.9–5.2)	0.3 (0.1–1.0)
Depression	2.8 (1.5–4.9)	4.0 (3.0–5.4)	6.5 (3.9–10.8)	1.1 (0.4–2.7)	0.2 (0.1–0.9)

**Table 2:** Comparison of older people with disabilities (‘cases’) and without disabilities (‘controls’) in sociodemographic characteristics.

	Cameroon			Guatemala			India			Haiti			Maldives			Nepal		
	Cases	Controls	OR ^1^ (95% CI)	Cases	Controls	OR ^1^ (95% CI)	Cases	Controls	OR ^1^ (95% CI)	Cases	Controls	OR ^1^ (95% CI)	Cases	Controls	OR ^1^ (95% CI)	Cases	Controls	OR ^1^ (95% CI)
Number:	104	153		210	78		125	136		47	26		119	97		86	84	
Age (years)																		
60–69	25%	34%	1	38%	55%	1	49%	65%	1	45%	74%	1	34%	46%	1	49%	47%	1
70–79	38%	40%	1.3 (0.7–2.6)	33%	37%	1.3 (0.7–2.3)	28%	23%	1.6 (0.5–5.2)	37%	23%	2.6 (1.5–4.5)	42%	32%	1.7 (0.9–3.3)	24%	35%	0.7 (0.4–1.5)
80+	36%	26%	2.0 (1.0–3.8)	29%	8%	5.5 (2.2–3.8)	23%	12%	3.0 (0.7–12.6)	16%	4%	7.6 (2.7–21.5)	25%	22%	1.5 (0.7–3.1)	28%	17%	1.7 (0.8–3.8)
Sex								Sex										
Male	44%	41%	1	47%	45%	1	34%	31%	1	40%	51%	1	49%	44%	1	51%	55%	1
Female	56%	59%	1.0 (0.6–1.6)	53%	55%	1.0 (0.6–1.7)	66%	69%	0.7 (0.2–2.9)	60%	49%	1.6 (0.9–2.7)	51%	56%	0.9 (0.5–1.5)	49%	45%	1.2 (0.6–2.2)
Socioeconomic position																		
1st (poorest)	25%	29%	1	25%	27%	1	18%	25%	1	23%	30%	1	26%	34%	1	47%	35%	1
2nd	25%	29%	0.9 (0.5–1.9)	27%	32%	0.8 (0.3–1.6)	27%	21%	1.2 (0.2–5.9)	25%	26%	1.5 (0.8–3.1)	25%	29%	1.2 (0.6–2.6)	21%	29%	0.5 (0.2–1.1)
3rd	26%	23%	1.3 (0.6–2.6)	24%	19%	0.7 (0.4–1.6)	33%	17%	2.6 (0.5–12.5)	25%	23%	1.6 (0.8–3.3)	22%	21%	1.6 (0.7–3.6)	16%	26%	0.5 (0.2–1.1)
4th (richest)	23%	18%	1.6 (0.7–3.3)	25%	22%	1.1 (0.5–2.5)	22%	38%	0.7 (0.2–3.1)	26%	21%	1.4 (0.7–2.9)	27%	16%	2.6 (1.2–5.9)	16%	11%	1.2 (0.5–3.2)
Education																		
No formal education	81%	84%	1	57%	55%	1	41%	40%	1	90%	83%	1	21%	26%	1	83%	75%	1
At least some education	19%	16%	1.4 (0.7–2.9)	43%	45%	0.5 (0.2–1.6)	59%	60%	0.7 (0.2–2.1)	10%	17%	0.5 (0.2–1.2)	79%	74%	0.8 (0.4–1.5)	17%	25%	0.7 (0.3–1.8)
Literacy									Literacy									
Literate	26%	18%	1	95%	96%	1	72%	80%	1	12%	23	1	84%	93%	1	24%	39%	1
Illiterate	74%	82%	0.5 (0.3–1.0)	5%	4%	1.0 (0.5–1.8)	27%	20%	0.6 (0.5–2.6)	88%	76%	2.0 (1.0–4.3)	16%	7%	2.4 (1.0–6.2)	76%	61%	2.1 (0.9–4.7)
Marital status																		
Married	66%	64%	1	37%	36%	1	33%	32%	1	70%	81%	1	59%	60%	1	61%	74%	1
Widowed/divorced	35%	33%	1.0 (0.6–2.0)	20%	24%	1.0 (0.5–1.8)	41%	44%	0.7 (0.2–4.0)	30%	18%	1.2 (0.6–2.5)	40%	40%	1.0 (0.5–1.8)	34%	25%	1.6 (0.8–3.3)
Not married	2%	3%	*	43%	40%	0.8 (0.2–2.9)	26%	24	1.3 (0.2–4.0)	0%	1%	*	1 (0.8%)	0%	*	6%	1%	*

^1^ Adjusted for age and sex using logistic regression; * cell sizes too small to calculate odds ratios. Cases = older people with disabilities; Controls = older people without disabilities.

**Table 3:** Comparison of older people with disabilities (‘cases’) and without disabilities (controls) in hygiene, health and healthcare and work.

	Cameroon			Guatemala			Haiti			India			Maldives			Nepal		
	Cases	Controls	OR ^1^ (95% CI)	Cases	Controls	OR ^1^ (95% CI)	Cases	Controls	OR ^1^ (95% CI)	Cases	Controls	OR ^1^ (95% CI)	Cases	Controls	OR ^1^ (95% CI)	Cases	Controls	OR ^1^ (95% CI)
Number:	104	153		210	78		47	26		125	136		119	97		86	84	
Water and Sanitation																		
Same toilet facility as other household members	-	-	-	90%	99%	0.2 (0.1–1.2)	-	-	-	-	-	-	-	-	-	83%	93%	0.4 (0.1–1.1)
Use facility without faecal contact	-	-	-	65%	69%	0.8 (0.4–1.5)	-	-	-	-	-	-	-	-	-	88%	97%	0.2 (0.04–1.0)
Use facility without assistance	-	-	-	70%	77%	0.6 (0.3–1.3)	-	-	-	-	-	-	-	-	-	77%	96%	0.1 (0.04–0.5)
Healthcare																		
Health problems and access to healthcare																		
Serious health problem last 12 months	52%	45%	1.3 (0.8–2.2)	48%	23%	4.2 (2.3–7.8)				26%	22%	1.6 (0.9–2.9)	-	-	-	17%	13%	1.4 (0.6–3.2)
Sought advice when had health problem	82%	88%	0.5 (0.2–1.5)	77%	78%	1.1 (0.3–3.8)				80%	85%	0.6 (0.07–5.8)	-	-	-	100%	100%	*
Tested for diabetes				32%	38%	1.0 (0.5–1.7)	-	-	-	-	-	-	-	-	-	33%	22%	1.4 (0.7–2.8)
Tested for hypertension				67%	56%	1.7 (0.9–2.9)	-	-	-	-	-	-	-	-	-	69%	73%	0.8 (0.4–1.7)
Diagnosis of diabetes and hypertension																		
Diagnosed with diabetes	-	-	-	17%	14%	1.8 (0.8–3.9)		-	-	-	-	-	23%	22%	1.0 (0.5–2.0)	4%	3%	0.8 (0.2–4.7)
Diagnosed with hypertension	-	-	-	36%	22%	2.4 (1.3–4.4)		-	-	-	-	-	61%	45%	1.9 (1.1–3.3)	45%	44%	0.9 (0.5–1.8)
Work ^2^																		
Work past week/current work	35%	58%	0.4 (0.2–0.7)	18%	44%	0.4 (0.2–0.7)	11%	34%	0.2 (0.1–0.9)	18%	52%	0.3 (0.2–0.5)	15%	27%	0.5 (0.3–1.1)	11%	30%	0.3 (0.1–0.6)
Work past year	58%	82%	0.3 (0.2–0.6)	26%	52%	0.5 (0.3–0.9	19%	42%	0.4 (0.1–1.1)	22%	56%	0.3 (0.2–0.5)	19%	35%	0.4 (0.2–0.8)	16%	36%	0.3 (0.1–0.7)

Cases = older people with disabilities; Controls = older people without disabilities. ^1^ Adjusted for age and sex using logistic regression; ^2^ in Cameroon and India, ‘work’ included work on a participant’s own land, while in the other settings it did not include this; * cell sizes too small to calculate odds ratios; - data not collected in this setting.

**Table 4:** Proportion of older adults with and without disabilities reporting that different aspects of the environment were a ‘big problem’ in terms of creating a barrier to participation in activities that matter to them.

Aspect of environment	Cameroon			Guatemala			Haiti			India		
	Cases	Controls	OR ^1^ (95% CI)	Cases	Controls	OR ^1^ (95% CI)	Cases	Controls	OR ^1^ (95% CI)	Cases	Controls	OR ^1^ (95% CI)
Transport availability or accessibility	34%	22%	1.8 (1.0–3.2)	29%	14%	2.4 (1.2–5.0)	36%	27%	1.3 (0.4–4.2)	38%	22%	2.0 (1.1–3.6)
Natural environnement (terrain, climate)	34%	15%	3.0 (1.6–5.4)	25%	9%	3.6 (1.5–8.4)	26%	19%	1.4 (0.4–4.7)	18%	16%	1.1 (0.5–2.2
Surroundings (lighting, noise, crowds)	8%	6%	1.4 (0.5–3.6)	23%	9%	3.1 (1.3–7.2)	17%	12%	*	13%	12%	0.9 (0.4–1.9)
Format of information	4%	3%	1.3 (0.3–4.9)	19%	9%	2.2 (0.9–5.1)	12%	4%	*	15%	8%	2.0 (0.9–4.5)
Availability of healthcare services	20%	19%	1.0 (0.5–1.8)	35%	28%	1.4 (0.8–2.5)	35%	16%	*	30%	17%	1.9 (1.1–3.7)
Availability of assistance at home	16%	7%	2.8 (1.2–6.4)	18%	15%	1.4 (0.7–2.9)	11%	17%	*	38%	11%	5.2 (2.6–10.9)
Availability of assistance at work	4%	0%	*	12%	3%	6.2 (1.4–27.5)			*	10%	10%	1.2 (0.5–2.8)
Other people’s attitudes (at home)	5%	3%	*	10%	9%	1.5 (0.6–3.8)	16%	11%	*	17%	11%	1.7 (0.8–3.7)
Other people’s attitudes (at school/work)	4%	1%	*	6%	3%	3.8 (0.8–17.7)			*	7%	7%	1.0 (0.4–2.7)
Prejudice and discrimination	4%	1%	*	12%	12%	1.2 (0.5–2.7)	11%	8%	*	12%	5%	3.3 (1.3–8.8)
Policies and rules (Organisations)	2%	1%	*	2%	3%	1.3 (0.2–6.9)	0%	0%	*	8%	5%	-
Government programmes and policies	3%	2%	*	6%	4%	1.5 (0.4–5.7)	13%	12%	1.2 (0.2–8.6)	24%	16%	1.8 (10.9–3.2)

^1^ Controlled for age (as a continuous variable) and sex; * odds ratios not calculated due to small numbers. Cases = older people with disabilities; Controls = older people without disabilities.

## Data Availability

We are unable to make the databases publicly available as we did not have participant consent for this. We can, however, share the databases with researchers upon request.

## Supplementary Materials

The following are available online at https://www.mdpi.com/article/10.3390/ijerph18136962/s1, Table S1. Details of surveys included in this secondary data analysis.
