# Supplementary material for: Disability among Older People: Analysis of Data from Disability Surveys in Six Low- and Middle-Income Countries
Source: Int J Environ Res Public Health. 2021 Jun 29;18(13):6962. doi: 10.3390/ijerph18136962 (PMC8297350; doi:10.3390/ijerph18136962)
Supplement: Supplementary file 1 [file ijerph-18-06962-s001.zip › ijerph-1235509-supplementary.pdf]

Table S1. Details of surveys included in this secondary data analysis.

Site	Year of Survey	Sampling Method	Total Participants (Number 60+ Years)	Tool and Definition of Disability ('Case')	Reporting Approach	Case Control Study: Matching Criteria for Selecting Controls
**Cameroon** Fundong Health District, West Cameroon	2011	Clusters: 51 clusters of 80 people selected using PPS; Households within clusters: modified compact segment sampling; Sampling Frame: Indian Census 2011	3567 (429)	Washington Group Extended Set on Functioning/Short Set Enhanced "A lot of difficulty" or "cannot do" in any of following domains: seeing, hearing, walking or climbing steps, understanding, being understood, remembering, concentrating, self-care, upper body strength, fine motor dexterity, and/or reporting "a lot" of anxiety/depression daily	Self-report	Age (+/- 5 years), sex, cluster
**Guatemala** National	2016	Clusters: 280 clusters of 50 people selected using PPS; Households within clusters: modified compact segment sampling; Sampling Frame: National Census 2002	13073 (1148)	Washington Group Extended Set on Functioning/Short Set Enhanced "A lot of difficulty" or "cannot do" in any of following domains: seeing, hearing, walking or climbing steps, understanding, being understood, remembering, concentrating, self-care, upper body strength, fine motor dexterity, and/or reporting "a lot" of anxiety/depression daily	Self-report	Age (+/- 10 years), sex, cluster

**India** Northern half of Mahbubnagar District, Telangana State	2012	Clusters: 51 clusters of 80 people selected using PPS; Households within clusters: modified compact segment sampling; Sampling Frame: Indian Census 2011	3543 (352)	Self-report	Age (+/- 5 years), sex, cluster
**Maldives** National	2017	Clusters: 52 clusters of 1250 people selected using PPS; Households within clusters: modified compact segment sampling; Sampling Frame: Maldives Census 2014	5513 (449)	Self-report or proxy report if participant unavailable after 3+ attempts	Age (+/- 5 years), sex, cluster
**Nepal** Tanahun, Province No 4	2016	Clusters: 30 clusters of 200 people selected using PPS; Households within clusters: modified compact segment sampling;	6162 (915)	Self-report or proxy report if participant unavailable	Age (+/- 5 years), sex, cluster

		Sampling Frame: National Population and Housing Census 20011				
**Haiti** Port au Prince	2011	Clusters: 60 clusters of 50 people selected using PPS; Households within clusters: modified compact segment sampling; Sampling Frame: National Population and Housing Census of Haiti from 2003	3132 (206)	WG Short Set “Some difficulty” in at least 2 domains, or “a lot of difficulty/can’t” in at least 1 domain: seeing, hearing, mobility, cognition, self-care, communication	Household heads provided proxy-report, then cases confirmed with self-report	Age (+/- 3 years), sex, cluster
